# An analysis of temporal and generational trends in the incidence of anal and other HPV-related cancers in Southeast England

**DOI:** 10.1038/sj.bjc.6604871

**Published:** 2009-01-20

**Authors:** D Robinson, V Coupland, H Møller

**Affiliations:** 1 Thames Cancer Registry, Division of Cancer Studies, King's College London, 1st Floor, Capital House, Weston Street, London SE1 3QD, UK

**Keywords:** human papillomavirus, anogenital cancers, cohort effects

## Abstract

Patients diagnosed in 1960–2004 with cancer of the cervix, anus, vulva, vagina or penis were identified from the Thames Cancer Registry database, and age-standardised period (temporal) incidence rates calculated by direct standardisation. Age-cohort modelling techniques were used to estimate age-specific incidence rates in the earlier and later cohorts, enabling the calculation of age-standardised cohort (generational) rates. Incidence of anal cancer increased for both men and women over the period studied, mainly in those born from 1940 onwards. Similar generational patterns were seen for cancers of the vulva and vagina, but those for penile cancer were different. For cervix cancer, the steep downward trend in cohort rates due to screening levelled off in women born from 1940 onwards. Our findings are compatible with the hypothesis that changes in sexual practices were a major contributor to the increases of these cancers. Programmes of vaccination against HPV, aimed at reducing the burden of cervical cancer, may also help to reduce the incidence of cancer at other anogenital sites.

Human papillomavirus (HPV), a sexually transmitted pathogen infecting the anogenital epithelium, is the major cause of cervix cancer. In cervical cancer, HPV infection is the major cause (Schiffman *et al*, 1993; International Agency for Research on Cancer, 1995; zur Hausen, 1996; Walboomers *et al*, 1999; Schlecht *et al*, 2001; Bosch *et al*, 2002; Schiffman *et al*, 2007; Bosch and de Sanjosé, 2003), and positive associations have been found between HPV infection and cancers of the anus (Daling and Sherman, 1992; Frisch *et al*, 1997; Daling *et al*, 2004; Bjørge *et al*, 2002), vulva and vagina (Madeleine *et al*, 1997; Daling *et al*, 2002; Goffin *et al*, 2006; Madsen *et al*, 2008). Cancers of the anus, vulva and vagina occur more frequently than expected following cervical intraepithelial neoplasia III or invasive cervical cancer, suggesting a strong common aetiological factor (Evans *et al*, 2003). HPV infection may also play a role in a subset of penile squamous cell carcinomas (Iwasawa *et al*, 1993; Rubin *et al*, 2001; Daling *et al*, 2005; Heideman *et al*, 2007; Tornesello *et al*, 2008).

Increasing trends in anal cancer have been reported, possibly due to changes in sexual practices (Daling *et al*, 1987; Scholefield *et al*, 1990; Frisch *et al*, 1993, 1997). If this were the case, a strong generational effect would be expected in the incidence trends of anogenital cancers. We have explored this hypothesis by plotting age-standardised period (temporal) and cohort (generational) rates for cancer sites linked to HPV infection.

## Methods

Patients diagnosed in 1960–2004 with cancer of the anus (ICD-10 code C21), vulva (C51), vagina (C52), cervix uteri (C53) or penis (C60) were identified from the Thames Cancer Registry (TCR) database. TCR is a population-based registry, which in this period covered a population of approximately 12 million residents of London and southeast England. All tumours are coded according to the ICD-O classification (World Health Organization, 1976), and were retrospectively recoded to the ICD-10 classification in the case of tumours diagnosed before its introduction in the early 1990s. Examination of proportional frequency plots revealed no coding discrepancies or inconsistencies over the period of the study. Age at diagnosis was categorised into 5-year groups (0–4, 5–9, …, 80–84, 85+), and period of diagnosis into 5-year periods (1960–1964, 1965–1969, …, 2000–2004). Nominal birth cohort groups (1875, 1880, …, 2000) were then calculated by subtracting the midpoint of the age group from the midpoint of the period. For each site, an age-standardised incidence rate for each calendar period was calculated by direct standardisation, using the European standard population. As the earlier and later birth cohorts were missing data from the younger and older age groups, respectively, a Poisson regression age-cohort modelling technique (Clayton and Schifflers, 1987) was used to estimate the unavailable rates, enabling the estimation of an age-standardised incidence rate for each cohort. As estimates for later cohorts (represented by younger patients diagnosed in recent periods) were based on small numbers of cases, and hence relatively unreliable, we report only estimates for birth cohorts between 1885 and 1960.

## Results

Table 1 shows the total number of cancers diagnosed over the study period and the incidence by age for each site. Incidence increases with age at all sites, with few cases (other than cervical cancer cases) occurring before age 55.

**Figure 1:**
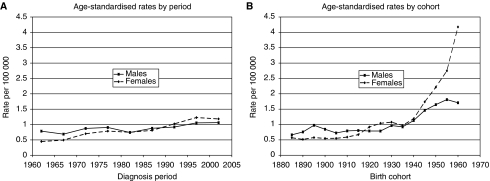
Anal cancer. (**A**) Age-standardised rates by period. (**B**) Age-standardised rates by cohort.

Figure 1A shows a modest increase in age-standardised period rates for anal cancer in both men and women. In men, these increased from 0.79 in 1960–64 to 1.06 per 100 000 in 2000–2004. In women, the increase was greater, from 0.45 in 1960–64 to 1.18 per 100 000 in 2000–2004. In both sexes, rates for consecutive cohorts (Figure 1B) remained relatively constant (between 0.5 and 1.1 per 100 000) for individuals born between 1885 and 1935. There was then a sharp increase in rates in the subsequent generations born from 1940 onwards. In men, the rates increased from 0.92 in the 1935 cohort to 1.71 per 100 000 in the 1960 cohort, whereas in women the increase was from 0.97 in the 1935 cohort to 4.18 per 100 000 in the 1960 cohort.

**Figure 2:**
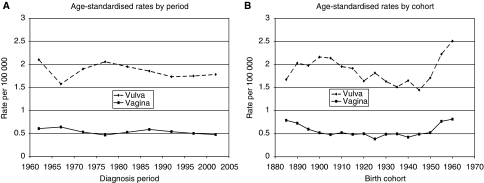
Vulval and vaginal cancers. (**A**) Age-standardised rates by period. (**B**) Age-standardised rates by cohort.

**Figure 3:**
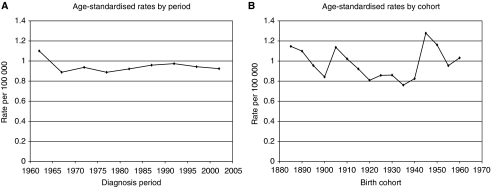
Penile cancer. (**A**) Age-standardised rates by period. (**B**) Age-standardised rates by cohort.

Figure 2A and B show age-standardised rates, by period and cohort, respectively, for cancers of the vulva and vagina. Period rates have remained fairly constant since the early 1990s, whereas cohort rates have increased in recent generations: for vaginal cancer, from 0.42 in the 1940 cohort to 0.82 per 100 000 in the 1960 cohort; the corresponding rise for vulval cancer was from 1.65 to 2.51. For penile cancer, period rates have remained fairly constant, whereas cohort rates have been erratic, with no clear pattern emerging (Figure 3A and B).

**Figure 4:**
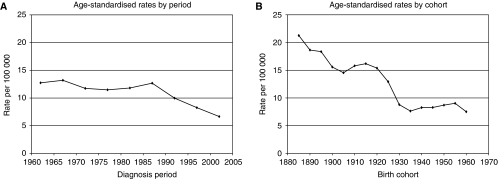
Cervical cancer. (**A**) Age-standardised rates by period. (**B**) Age-standardised rates by cohort.

Figure 4A and B show age-standardised period and cohort rates for cervical cancer. Period rates have shown a dramatic decline from 12.68 in those diagnosed between 1985 and 1990 to 6.66 per 100 000 in 2000–04. Cohort rates declined from 21.27 in the 1885 cohort to 7.64 per 100 000 in the 1935 cohort, after which they remained relatively constant. Table 2 summarises these results, showing the age-standardised rates and associated 95% confidence levels.

## Discussion

Anal cancer incidence in southeast England increased in both men and women over the study period (1960–2004), by threefold in women and about 1.5-fold in men; the incidence is now higher in women than in men. Similar trends have been seen in Denmark (Frisch *et al*, 1993), the United States of America (Melbye *et al*, 1994; Johnson *et al*, 2004) and Sweden (Goldman *et al*, 1989). Increases have also been reported in Scotland (Brewster and Bhatti, 2006), where the change in male rates has roughly paralleled that in the female rates from the mid-1970s to the mid-1990s. Male rates have since plateaued, whereas female rates have continued to rise. In Scotland the female rates were higher than the males rates throughout the period studied, whereas in our population these were initially lower than in men, but became higher from the early 1990s onwards.

Judson *et al* (2006) found a 20% increase in invasive vulval cancer in the United States of America between 1973 and 2000. No increase was seen in the period rates for vulval cancer in our data, nor any increase in the period rates for cancers of the vagina or penis.

The dramatic decline in rates for cervical cancer from the late 1980s onward is similar to that reported in the West Midlands (Clare *et al*, 2008), and reflects the introduction of the National Health Service's screening programme around 1988, which has led to the early detection of precancerous cervical lesions and the prevention of many invasive cancers.

In our study, in both sexes a marked increase was found in the cohort rates for anal cancer in those born from around 1940 onwards. Similar generational patterns were seen for cancers of the vulva and vagina, consistent with a common aetiology. The most likely causes are changes in sexual practice and greater exposure to infectious agents such as HPV. For cancer of the cervix, for which HPV is generally recognised as the major cause, the steep downward trend in cohort rates due to screening levelled off in women born from 1940 onwards.

We found no clear-cut change in cohort rates for penile cancer, although on average the rates for generations born after 1940 were higher than in previous cohorts. It may be that the effect of HPV infection is less in penis cancer than in the other cancers, as recently suggested (Tornesello *et al*, 2008).

Several studies have linked sexual behaviour with cancer of the anus (Daling *et al*, 1987; Scholefield *et al*, 1990; Frisch *et al*, 1993, 1997). In both Denmark (Melbye and Biggar, 1992) and Scotland (Brewster and Bhatti, 2006) a higher prevalence of receptive anal intercourse in women than in men has been reported, and may explain in part the higher incidence of anal cancer in women in these countries. Sexual practices have also been implicated in cancers of the vagina and vulva (Sherman *et al*, 1991; Madsen *et al*, 2008) and of the penis (Maden *et al*, 1993).

It should be noted that the cohorts which show the recent increases in incidence are, of necessity, based partially on modelled data, because observational data in the older age groups are not available. However, the variability introduced by the modelling procedure has been included in the calculation of the standard errors of the estimated rates. As can be seen from Table 2, none of the estimates exhibits excessive variation.

The generational patterns seen in the cohort rates presented in this paper, with a marked change from around 1940, while not constituting proof, are compatible with the hypothesis that changes in sexual practices are a major contributor to the increases in anogenital cancers other than the cervix. Those born around 1940 would have been in their early twenties at the start of the ‘sexual revolution’ in the early 1960s.

Peto *et al* (2004) have estimated that ‘cervical cancer screening has prevented an epidemic that would have killed about one in 65 of all British women born since 1950 and culminated in 6000 deaths per year in this country’. Trials of vaccination against HPV in teenage girls are currently underway in the United Kingdom. In Australia, it has been predicted that the public vaccination programme begun in 2007 will result in a reduction in the age-standardised incidence of HPV-16 infections of 56% by 2010 and 92% by 2050 (Smith *et al*, 2008). Programmes of vaccination, aimed at reducing the burden of cervical cancer, may also help to reduce the incidence of cancers of the vulva, vagina and anus in women.

## Figures and Tables

**Table 1:** Cancer incidence rates per 100 000 by age at diagnosis

	**Age**							
**Cancer site**	**<35**	**35–44**	**45–54**	**55–64**	**65–74**	**75–84**	**85+**	**Total cancers diagnosed**
Anus, males	0.02	0.39	0.97	2.14	3.41	5.45	7.28	1988
Anus, females	0.03	0.44	1.18	1.95	3.37	4.75	6.60	2676
Vulva	0.09	0.74	1.46	3.15	7.44	14.24	22.86	6216
Vagina	0.04	0.24	0.66	1.06	1.80	3.46	4.19	1639
Penis	0.04	0.46	0.94	1.92	3.49	6.08	10.24	2082
Cervix	2.93	16.21	18.34	19.89	20.25	20.34	19.43	25854

**Table 2:** Age-standardised incidence rates (per 100,000) and 95% confidence intervals

**Site**	**Anus, males**	**Anus, females**	**Vulva**	**Vagina**	**Penis**	**Cervix**
*Period*						
1962	0.79 (0.64–0.93)	0.45 (0.36–0.54)	2.10 (1.91–2.29)	0.60 (0.50–0.70)	1.10 (0.93–1.27)	12.74 (12.23–13.25)
1967	0.69 (0.56–0.82)	0.50 (0.40–0.59)	1.57 (1.41–1.73)	0.64 (0.53–0.74)	0.89 (0.74–1.04)	13.20 (12.67–13.73)
1972	0.87 (0.73–1.02)	0.70 (0.59–0.81)	1.90 (1.72–2.08)	0.53 (0.43–0.62)	0.94 (0.78–1.09)	11.74 (11.24–12.24)
1977	0.91 (0.76–1.05)	0.79 (0.67–0.91)	2.05 (1.87–2.24)	0.46 (0.37–0.55)	0.89 (0.74–1.04)	11.48 (10.97–11.99)
1982	0.74 (0.61–0.88)	0.75 (0.63–0.87)	1.95 (1.77–2.13)	0.52 (0.43–0.62)	0.92 (0.77–1.07)	11.81 (11.29–12.33)
1987	0.88 (0.78–0.98)	0.81 (0.72–0.90)	1.85 (1.73–1.98)	0.58 (0.51–0.66)	0.96 (0.85–1.07)	12.68 (12.30–13.06)
1992	0.92 (0.81–1.02)	1.02 (0.92–1.12)	1.73 (1.61–1.85)	0.54 (0.46–0.61)	0.97 (0.87–1.08)	9.98 (9.65–10.32)
1997	1.05 (0.94–1.16)	1.23 (1.12–1.34)	1.75 (1.62–1.87)	0.50 (0.43–0.57)	0.94 (0.84–1.05)	8.26 (7.96–8.55)
2002	1.06 (0.95–1.17)	1.18 (1.08–1.29)	1.78 (1.65–1.90)	0.47 (0.41–0.54)	0.92 (0.82–1.02)	6.66 (6.40–6.92)
						
*Cohort*						
1885	0.66 (0.55–0.77)	0.57 (0.50–0.64)	1.68 (1.56–1.79)	0.79 (0.70–0.88)	1.15 (1.00–1.30)	21.27 (20.40–22.13)
1890	0.76 (0.64–0.87)	0.52 (0.45–0.58)	2.03 (1.90–2.17)	0.73 (0.65–0.81)	1.10 (0.96–1.24)	18.65 (18.01–19.29)
1895	0.98 (0.84–1.11)	0.58 (0.50–0.65)	1.98 (1.84–2.11)	0.60 (0.52–0.67)	0.96 (0.82–1.09)	18.36 (17.81–18.91)
1900	0.85 (0.73–0.96)	0.55 (0.47–0.62)	2.17 (2.02–2.31)	0.52 (0.45–0.59)	0.84 (0.73–0.96)	15.58 (15.13–16.03)
1905	0.72 (0.62–0.83)	0.55 (0.47–0.63)	2.14 (1.99–2.29)	0.48 (0.41–0.55)	1.14 (1.00–1.27)	14.56 (14.15–14.97)
1910	0.79 (0.68–0.90)	0.59 (0.51–0.67)	1.96 (1.82–2.10)	0.52 (0.44–0.60)	1.02 (0.90–1.15)	15.80 (15.35–16.25)
1915	0.81 (0.69–0.92)	0.67 (0.58–0.76)	1.92 (1.77–2.07)	0.49 (0.40–0.57)	0.92 (0.80–1.04)	16.19 (15.70–16.67)
1920	0.79 (0.68–0.90)	0.92 (0.81–1.02)	1.64 (1.51–1.78)	0.50 (0.42–0.58)	0.81 (0.70–0.92)	15.39 (14.89–15.89)
1925	0.79 (0.69–0.89)	1.04 (0.93–1.16)	1.82 (1.67–1.97)	0.39 (0.32–0.45)	0.86 (0.75–0.97)	12.95 (12.47–13.43)
1930	0.97 (0.85–1.08)	1.08 (0.97–1.19)	1.64 (1.50–1.78)	0.49 (0.41–0.57)	0.86 (0.76–0.96)	8.80 (8.38–9.22)
1935	0.92 (0.82–1.03)	0.97 (0.87–1.07)	1.51 (1.39–1.64)	0.50 (0.42–0.57)	0.76 (0.67–0.85)	7.64 (7.26–8.03)
1940	1.13 (1.02–1.24)	1.19 (1.07–1.30)	1.65 (1.52–1.78)	0.42 (0.36–0.49)	0.83 (0.73–0.92)	8.28 (7.90–8.66)
1945	1.46 (1.32–1.60)	1.74 (1.59–1.89)	1.45 (1.33–1.57)	0.49 (0.42–0.56)	1.28 (1.16–1.40)	8.29 (7.96–8.63)
1950	1.65 (1.48–1.82)	2.22 (2.01–2.43)	1.72 (1.57–1.86)	0.52 (0.45–0.59)	1.16 (1.04–1.28)	8.72 (8.42–9.03)
1955	1.81 (1.59–2.04)	2.75 (2.43–3.06)	2.23 (2.03–2.42)	0.77 (0.66–0.88)	0.95 (0.83–1.07)	9.05 (8.76–9.34)
1960	1.71 (1.44–1.98)	4.18 (3.60–4.75)	2.51 (2.26–2.77)	0.82 (0.67–0.96)	1.03 (0.88–1.18)	7.52 (7.29–7.75)
